# The Management of Hyperplastic Pulpitis in Immature Permanent Molar Using Vital Pulp Therapy: A Case Report with 12 Months Follow-Up

**DOI:** 10.1155/2024/5280168

**Published:** 2024-06-10

**Authors:** Anas Mando, Mohannad Laflouf, Yasser Alsayed Tolibah

**Affiliations:** Department of Pediatric Dentistry Damascus University, Damascus, Syria

## Abstract

Hyperplastic pulpitis is an irreversible type of pulpitis that primarily affects young patients. It occurs when an inflamed pulp becomes exposed due to factors such as dental caries, dental trauma, or other causes. Root canal treatment is commonly employed to manage hyperplastic pulpitis. However, vital pulp therapy can be considered as a less invasive option. The main objective of this treatment is to preserve the vitality and functionality of the remaining pulp tissue. This case report discusses the potential management of hyperplastic pulpitis in an immature molar using vital pulp therapy instead of a full root canal treatment. The report includes clinical and radiographic follow-up at six and twelve months.

## 1. Introduction

Pulpitis is a common dental condition characterized by inflammation of the dental pulp. It can be caused by various factors, including dental decay, tooth trauma, or other dental procedures [1]. According to the American Association of Endodontists, hyperplastic pulpitis, also known as pulp polyp, is a type of chronic pulpal inflammation that often develops after carious or traumatic exposure. It is characterized by the proliferation of dental pulp tissue from the exposed pulp chamber, which fills the cavity with a pedunculated or sessile, pinkish-red, fleshy mass, typically covered with epithelium [2]. The primary and immature permanent molars are the most commonly affected teeth, and the condition may be asymptomatic or cause pressure during biting [3]. The absence of a roof in the pulp chamber and the presence of open root apices can reduce intrapulpal pressure, maintain microcirculation, and decrease the likelihood of pulpal necrosis [3].

Vital pulp therapy (VPT) is a conservative treatment option aimed at preserving the vitality and functionality of the remaining pulp tissue [4]. This treatment has high success rates in both primary and permanent teeth. Furthermore, in immature molars, VPT can effectively save a decayed tooth without resorting to more invasive procedures like extraction or root canal therapy [5]. VPT becomes particularly important when managing vital immature teeth with deep caries and open apices, as these teeth are still in the development process and more prone to future fractures [6].

While root canal therapy is commonly used to treat hyperplastic/irreversible pulpitis, it poses challenges when dealing with teeth that have open apices and are still developing [7, 8]. Therefore, the objective of this case report is to manage the hyperplastic pulpitis in an immature molar through the use of full (cervical) pulpotomy as a form of VPT, using novel calcium silicate cement known as bioceramic putty.

## 2. Case Presentation

The project was ethically approved by the Local Research Ethics Committee of the Faculty of Dentistry, Damascus University (UDDS-2653-14022023/SRC-365).

**Figure 1:**
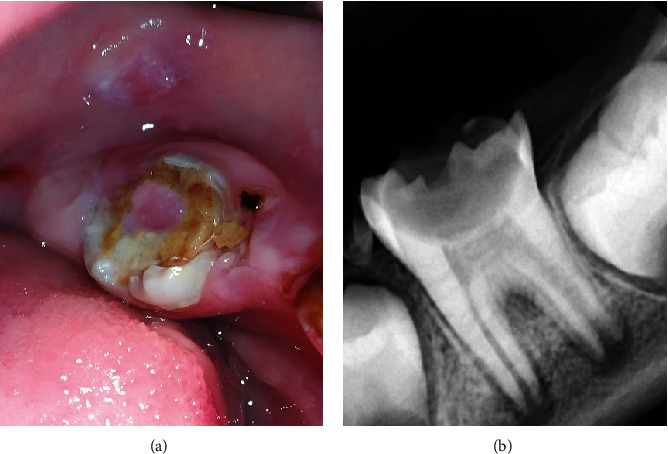
The lower left first permanent molar: (a) initial intraoral image and (b) preoperative periapical radiograph.

A 7-year-old female patient presented to the Department of Pediatric Dentistry with a chief complaint of spontaneous pain in the mandibular teeth. During the dental examination, sensitivity to cold stimulation and spontaneous pain were observed in the permanent first mandibular left molar. Radiographic and clinical examinations revealed a deep carious lesion penetrating the pulp (Figures 1(a) and 1(b)).

The parents were informed about the available treatment options, which included extraction, complete pulpectomy, or pulpotomy. The potential outcomes of each treatment option were thoroughly explained. After obtaining informed consent, the chosen treatment plan was cervical pulpotomy.

**Figure 2:**
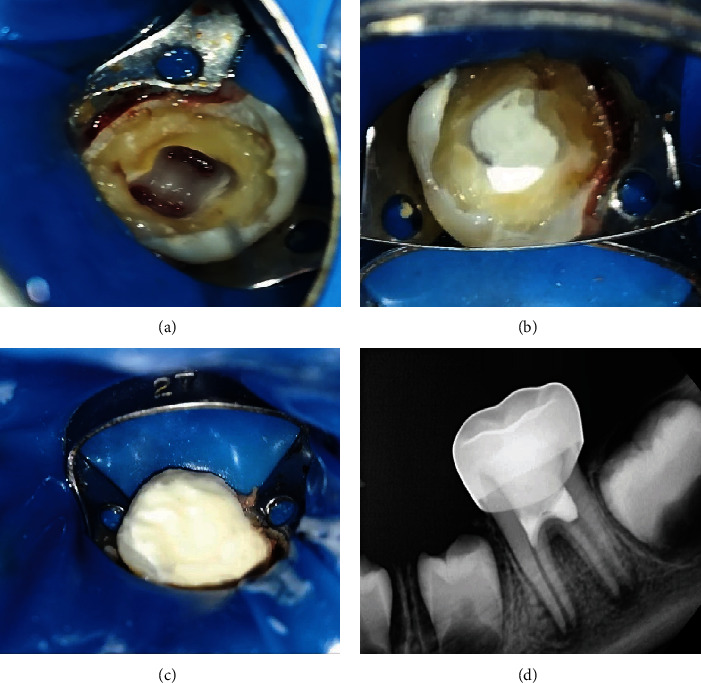
Pulpotomy procedures: (a) hemostasis achieved, (b) bioceramic putty application, (c) building coronal part with GIC, and (d) postoperative periapical radiograph.

**Figure 3:**
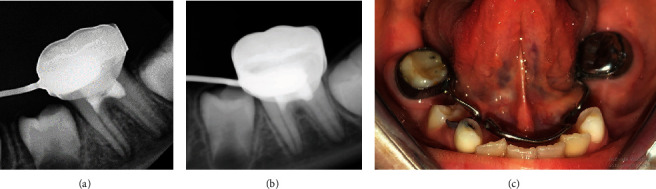
Follow-up images: (a) 6 months periapical radiograph, (b) 12 months periapical radiograph, and (c) 12 months final intraoral image.

Under local anesthesia with 2% lidocaine and 1:100,000 epinephrine (Novocol Pharma, Ontario, Canada) and rubber dam isolation, soft and decayed tissues were removed using a low-speed handpiece with a round carbide bur (RA#6, Dentsply Sirona, Charlotte, North Carolina, USA). Subsequently, the access cavity was prepared with a diamond fissure bur (Cylinder Flat End - FG - Coarse – 016, Dentsply Maillefer, Ballaigues, Switzerland), and a Tungsten carbide bur (Endo-Z - Dentsply Maillefer, Ballaigues, Switzerland) was used at high-speed with copious water spray to prevent heat damage to the remaining radicular pulp. After accessing the pulp chamber, it was rinsed with saline, and a large spoon excavator was utilized to remove the pulp polyp tissue to the level of canal orifices. A wet cotton pellet soaked with 2% NaOCl was then applied for 4 minutes to control bleeding [2]. Once hemostasis was achieved (Figure 2(a)), bioceramic putty (Well-Root PT; Vericom, Chuncheon, Korea) was used (Figure 2(b)) and adapted onto the radicular pulp with a moist cotton pellet under light pressure. Finally, the access cavity was restored with glass ionomer cement (GC Fuji II, GC America, Chicago, Illinois, USA) (Figure 2(c)), and a stainless-steel crown (3M ESPE, St. Paul, MN, USA) was placed as the final restoration (Figure 2(d)). The patient was scheduled for follow-up at 6 and 12 months (Figure 3) after the treatment. The tooth showed normal function and was asymptomatic, with no clinically or radiographically evident signs of necrosis. Radiographic evidence demonstrated root development and formation of the root apex during the follow-up sessions.

## 3. Discussion

Preserving pulp vitality in young patients is crucial to support the growth and development of immature teeth [9]. Nevertheless, it is noted that among this age group, oral hygiene knowledge, awareness, and practices often fall below satisfactory levels [10]. Despite these challenges, efforts should be directed toward the early diagnosis, treatment, and preservation of the dental pulp. A range of treatment choices are available for addressing such cases, with the selection depending on factors like the severity of the condition, the patient's age, and the stage of root development [11]. Treatment modalities may encompass apexogenesis, apexification, regeneration, or extraction, each tailored to the specific needs of the individual case [12, 13].

Pulpotomy is a procedure that involves removing inflamed pulp tissue from the pulp chamber while leaving the healthy pulp tissue in the root canals untouched. Following this, a medicament may be applied, and the tooth receives a final restoration. This approach helps maintain the remaining healthy pulp tissue and encourages the continuation of root development [9, 14, 15].

Apexification entails placing a biocompatible material, such as mineral trioxide aggregate (MTA), at the root apex to stimulate the formation of a hard tissue barrier without fostering root development [16].

Regenerative endodontic procedures involve the removal of inflamed pulp tissue and the placement of a scaffold material to encourage the regeneration of new tissue. This treatment aids in preserving healthy pulp tissue, supporting ongoing root development, and potentially regenerating damaged pulp tissue [9]. A previous case study detailed a successful regenerative endodontic procedure on a necrotic immature mandibular molar using calcium hydroxide and triple antibiotic paste dressings [17].

Extraction is considered an alternative treatment for pediatric patients with one or more first permanent molars (FPM) with a poor prognosis. The primary goal is the successful eruption of the second permanent molar as a suitable replacement, with the ideal scenario involving the eruption of the third molar to complete the molar dentition. However, FPM extraction can result in the misalignment of adjacent teeth and an improper bite, necessitating orthodontic intervention and complicating treatment for both the child and parents [13].

In the present case, the initial diagnosis was hyperplastic pulpitis, commonly referred to as pulp polyp. Historically, extraction has been recommended as the preferred management option for teeth affected by this condition, particularly when significant dental tissue loss is present due to decay [18]. However, traditional endodontic treatments have been advocated for cases involving closed apex teeth [19]. Furthermore, partial pulpotomy with calcium hydroxide has been proposed, involving the removal of inflamed pulp tissue followed by the application of calcium hydroxide [20].

Recent studies have highlighted that vital pulp therapies extend beyond cases of reversible pulpitis. Hence, a diagnosis of “irreversible pulpitis” does not necessarily mandate pulpectomy [14, 15, 21]. Studies have reported success rates ranging from 78.1% to 100% for full pulpotomy in teeth with irreversible pulpitis. When periapical radiolucencies are present alongside irreversible pulpitis, success rates range from 65.7% to 100% [15].

In this instance, the selected treatment was a full (cervical) pulpotomy, chosen to minimize treatment sessions and maintain the child's cooperation. Additionally, a study has suggested that the outcomes of healing for this type of inflammation surpass those of chronic ulcerative pulpitis, indicating the favorable reparative abilities of pulp tissue due to the hyperplasia of granulation tissue [20].

A previous report highlighted a successful approach in managing chronic hyperplastic pulpitis in mature molars and canines, showcasing a high frequency of clinical healing. The technique involved excising only the polyp using a new sterile excavator (partial pulpotomy), controlling hemorrhage from the coronal pulp with saline solution and sterile cotton pellets, and covering the pulp with pure calcium hydroxide. Notably, a continuous hard tissue barrier was clinically observed in 20 teeth during recall examinations, and radiographic evaluations revealed dentin bridges in 10 cases [20].

These findings underpin the recommendation of vital pulp therapy (VPT) as a viable treatment option for specific cases of chronic hyperplastic pulpitis. Calcium silicate cements, particularly mineral trioxide aggregate (MTA), have shown superior histological and clinical outcomes compared to calcium hydroxide for managing exposed pulp [22–27]. A recent study comparing various pulp capping materials, including bioactive options like Biodentine, MTA, and bioceramic putty, revealed that bioactive materials are more favorable alternatives for pulp capping [28]. In this context, bioceramic putty was selected for cervical pulpotomy due to its ease of use and application as a premixed paste [29]. Furthermore, a prior histological investigation indicated that bioceramic putty offers comparable biocompatibility to MTA and can yield a thicker dentin barrier when covering pulp tissues compared to MTA [30].

On another note, a recent randomized controlled trial focusing on the treatment of immature molars with irreversible pulpitis utilized bioceramic and MTA. Following a one-year follow-up, bioceramic putty demonstrated a clinical and radiographic success rate of 93.4%, with only one case of failure. Meanwhile, MTA exhibited a 100% success rate both clinically and radiographically after a one-year evaluation period [31].

Hemostasis in the pulp tissue is commonly achieved by immersing the resected pulp tissue in sodium hypochlorite for 5 to 10 minutes. The recommended duration for this procedure may vary and can be carried out through direct dpassive irrigation or by placing a sodium hypochlorite-soaked cotton pellet [15]. Studies have confirmed that sodium hypochlorite, at various concentrations, can be safely used in direct contact with pulp tissue without compromising its integrity. Moreover, it effectively addresses aesthetic concerns by removing composite staining [15].

In endodontic procedures, the restoration of the tooth is pivotal, and immediate restoration should be a key element of the overall restorative treatment plan for teeth undergoing vital pulp therapy. The use of different calcium silicate cements as the primary sealing material, followed by immediate restoration, has demonstrated high success rates [15]. Notably, in cases where a significant portion of the coronal tissues are affected by caries, a full coverage restoration is essential to restore the structural integrity of the tooth.

Stainless steel crowns (SSCs) emerge as a cost-effective solution for the restoration of pediatric molars post vital pulp therapy. SSCs offer exceptional durability and are well-received by pediatric patients, providing long-term protection against fracture or recurrent decay in the treated tooth [9, 32]. In this case, a stainless steel crown would be a suitable choice for the final restoration of the treated tooth.

Further exploration and research into the treatment of hyperplastic pulpitis using innovative bioceramic materials with extended follow-up periods are recommended. These materials could also be evaluated in conjunction with recent advancements like low-noise instruments [33] and computerized anesthesia devices [34] in upcoming clinical trials. It should be acknowledged that the treatment methods and strategies discussed above are underpinned by general principles and may be subject to variations depending on the individual case and the clinical judgment of the dentist. It is advisable to seek guidance from a qualified dental professional who can assess the patient's specific condition and offer tailored treatment recommendations.

The limitations of this case report are the inability to use microscopic magnification, the inability to find more cases to make a case series, the inability to visually detect the formation of the calcified bridge, and the inability to perform pulp sensibility tests due to the presence of stainless-steel crown as a final restoration.

## 4. Conclusion

This case report suggests that vital pulp therapy with bioceramic putty pulpotomy can be a viable treatment option for managing hyperplastic pulpitis in immature molars, with favorable outcomes after 1 year of follow-up.

## Data Availability

Clinical trials data supporting this case report are from previously reported studies and datasets, which have been cited and can be found in references.
